# Supplementary material for: In silico analysis of local RNA secondary structure in influenza virus A, B and C finds evidence of widespread ordered stability but little evidence of significant covariation
Source: Sci Rep. 2022 Jan 10;12:310. doi: 10.1038/s41598-021-03767-x (PMC8748542; doi:10.1038/s41598-021-03767-x)
Supplement: Supplementary file 1 — Supplementary Information. [file 41598_2021_3767_MOESM1_ESM.zip › Supplemental Files/Supplemental File 3.zip!/Supplemental File 3/CM-AA-rev/pdf/IAV_seg1_2_1.fold.R2R.sto.pdf]

IAV_seg1_2_1

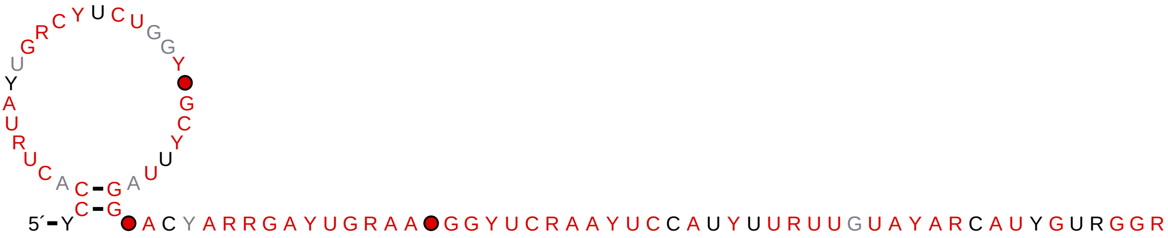
5´
Y
C C A C U R U A Y U G R C Y U C U G G Y G C Y U U A G G
A C Y A R R G A Y U G R A A G G Y U C R A A Y U C C A U Y U U R U U G U A Y A R C A U Y G U R G G R
